# The Popular Science Monthly

**Published:** 1877-07

**Authors:** 


### The Popular Science Monthly.

No publication has done so much to educate the people in true scientific principles of thought, as this magazine. All scientific discoveries of any value have been noted and discussed by the greatest scientists in Europe and America, so that by its aid a man of ordinary education can keep well posted upon the scientific events of the day. Not satisfied with the noble work occomplished, they astonished the reading world by publishing in May, a new issue, "The Popular Science Monthly Supplement," a monthly complete in itself, and of such interest and merit that it will be likely to advance the esteem in which the parent magazine is already held. Three numbers have already been published.

We would call the attention of our readers to the June number, in which will be found an article by Dr. Max Von Pettenkofer, "Relation of air to the house we live in," which presents in a masterly way the principle of ventilation. We publish an extract from this, in our present number. Of equal interest in a medical point of view, is article III, July number, by the same author, on ground air in its hygienic relations. But in such an array of literary thought we cannot particularize, and can only say, subscribe and investigate its rich fields for yourselves.

